# Changes in Testosterone Levels and Sex Hormone-Binding Globulin Levels in Extremely Obese Men after Bariatric Surgery

**DOI:** 10.1155/2016/1416503

**Published:** 2016-09-20

**Authors:** Patchaya Boonchaya-anant, Nitchakarn Laichuthai, Preaw Suwannasrisuk, Natnicha Houngngam, Suthep Udomsawaengsup, Thiti Snabboon

**Affiliations:** ^1^Hormonal and Metabolic Disorders Research Unit, Excellence Center for Diabetes, Hormone, and Metabolism, Department of Medicine, Faculty of Medicine, Chulalongkorn University, Bangkok, Thailand; ^2^Division of Endocrinology and Metabolism, Department of Medicine, Faculty of Medicine, Chulalongkorn University, Bangkok, Thailand; ^3^King Chulalongkorn Memorial Hospital, Thai Red Cross Society, Bangkok, Thailand; ^4^Department of Medicine, Faculty of Medicine, Naresuan University Hospital, Phitsanulok, Thailand; ^5^Department of Surgery, Faculty of Medicine, Chulalongkorn University, Bangkok, Thailand

## Abstract

*Objective.* Obesity is a risk factor for hypogonadotropic hypogonadism in men. Weight loss has been shown to improve hypogonadism in obese men. This study evaluated the early changes in sex hormones profile after bariatric surgery.* Methods.* This is a prospective study including 29 morbidly obese men. Main outcomes were changes in serum levels of total testosterone (TT), free testosterone (cFT), SHBG, estradiol, adiponectin, and leptin at 1 and 6 months after surgery.* Results.* The mean age of patients was 31 ± 8 years and the mean BMI was 56.8 ± 11.7 kg/m^2^. Fifteen patients underwent Roux-en-Y gastric bypass and 14 patients underwent sleeve gastrectomy. At baseline, 22 patients (75.9%) had either low TT levels (<10.4 nmol/L) or low cFT levels (<225 pmol/L). Total testosterone and SHBG levels increased significantly at 1 month after surgery (*p* ≤ 0.001). At 6 months after surgery, TT and cFT increased significantly (*p* ≤ 0.001) and 22 patients (75.9%) had normalized TT and cFT levels. There were no changes in estradiol levels at either 1 month or 6 months after surgery.* Conclusions*. Increases in TT and SHBG levels occurred early at 1 month after bariatric surgery while improvements in cFT levels were observed at 6 months after bariatric surgery.

## 1. Introduction

Obesity is now a worldwide epidemic [1]. Comorbidities related to obesity are numerous including reproductive disorder. Male obesity-associated secondary hypogonadism (MOSH) has been described in male obesity. MOSH is characterized by low circulating testosterone levels along with low or inappropriately normal FSH and LH levels in the absence of pituitary disease. Prevalence of MOSH has been reported up to 40–50% in obese men [2].

The exact mechanisms that cause hypogonadism in obese men are still not fully understood. Several processes have been observed in MOSH. First, low sex hormone-binding globulin (SHBG) levels seen in MOSH are similar to those with metabolic syndrome and type 2 diabetes [3] and decreased SHBG level is associated with the increase in body mass index (BMI) and insulin resistance [4, 5]. Secondly, increased estradiol levels and adipokines from an excess adipose tissue may have an effect on gonadotropins pulsatility and secretion [6]. Thirdly, leptin, the first discovered adipokine, increases in obesity and the level was found to be associated with the decrease in testosterone and SHBG levels [7, 8]. Additionally, circulating adiponectin, an adipokine that plays an important role in insulin sensitivity, is decreased with obesity [9]. Study in mice showed that adiponectin regulates gonadotropins secretion [10] although there is still no evidence in human.

Weight loss with diet and exercise increases insulin sensitivity and improves testosterone levels in obese men [11]. Bariatric surgery is an effective treatment for obesity. Several reports have shown improvement in testosterone levels after bariatric surgery at 6–24 months of follow-up [12–14]. In this study, we would like to examine the early changes in sex hormones profile after bariatric surgery during the acute weight loss and its relation to adipokines changes.

## 2. Subjects and Methods

We enrolled 36 obese males who were referred to bariatric and metabolic institute at King Chulalongkorn Memorial Hospital for weight loss surgery. Inclusion criteria were age ≥18 and meeting bariatric surgery criteria by The 1991 NIH Consensus Conference [15]. Exclusion criteria were previous diagnoses of hypogonadism, any use of testosterone products or treatment for hypogonadism, pituitary disease, chronic kidney disease, or chronic liver disease. The type of bariatric procedure was chosen by a multidisciplinary team as deemed appropriated for patients' body mass index (BMI) and comorbidities. Written informed consent was obtained and the study was approved by the Institutional Review Board of our hospital.

All patients underwent standard evaluation process for bariatric surgery and were assessed by an endocrinologist at baseline for signs and symptoms of hypogonadism. Anthropometric parameters were recorded at baseline and during follow-up visits. Morning venous blood draws after 12 h overnight fasting were obtained at baseline and at 1-month and 6-month visits after bariatric surgery. Serum samples were measured for total testosterone (TT), SHBG, and estradiol by electrochemiluminescent immunoassay (Elecsys kit; Roche Diagnostics) and adiponectin and leptin by ELISA (Millipore). Calculated free testosterone (cFT) was calculated from total testosterone, SHBG, and albumin using the Vermeulen equation [16].

Reference ranges of total testosterone, free testosterone, SHBG, and estradiol were provided by central laboratory of King Chulalongkorn Memorial Hospital. Normal ranges for men aged ≤40 years were 10.4–34.7 nmol/L for TT, 225–880 pmol/L for cFT, 14.5–48.4 nmol/L for SHBG, and 0–130 pmol/L for estradiol.

ADAM questionnaire was administered at baseline and at 6 months after bariatric surgery to screen for symptoms of hypogonadism.

*Statistical Analysis*. Statistical analysis was performed with the SPSS version 20.0 software (SPSS, Chicago, IL). Continuous variables were described as means ± SD or median (interquartile range). Categorical variables were reported as percent positive. Differences in continuous variables between pre- and postsurgery were assessed with paired *t*-test. Pearson correlation test was used to examine the relationship between two parameters. A value of *p* < 0.05 was assumed as statistically significant.

## 3. Results

Thirty-six obese male patients were enrolled in the study. One patient was excluded due to the diagnosis of prepubertal hypogonadism (incomplete development of male secondary sexual characteristics) and six patients did not complete the follow-up visits. Therefore, twenty-nine patients were included in the final analyses. Baseline characteristics are shown in Table 1. Seven patients (24.1%) had type 2 diabetes treated with oral antidiabetic drugs and only one patient was taking insulin. All 29 patients had obstructive sleep apnea.

At baseline, 16 patients (55.2%) had both low TT levels (<10.4 nmol/L) and low cFT levels (<225 pmol/L) and 6 patients (20.7%) had only low TT levels. Seven patients (24.1%) had both normal TT and cFT levels. At 6 months after surgery, 5 patients (17.2%) had both low TT and cFT levels, 6 patients (20.7%) had only low TT levels, and 7 patients (24.1%) had only low cFT levels. Twenty-two patients (75.9%) had both normal TT and cFT levels at 6-month follow-up.

**Figure 1:**
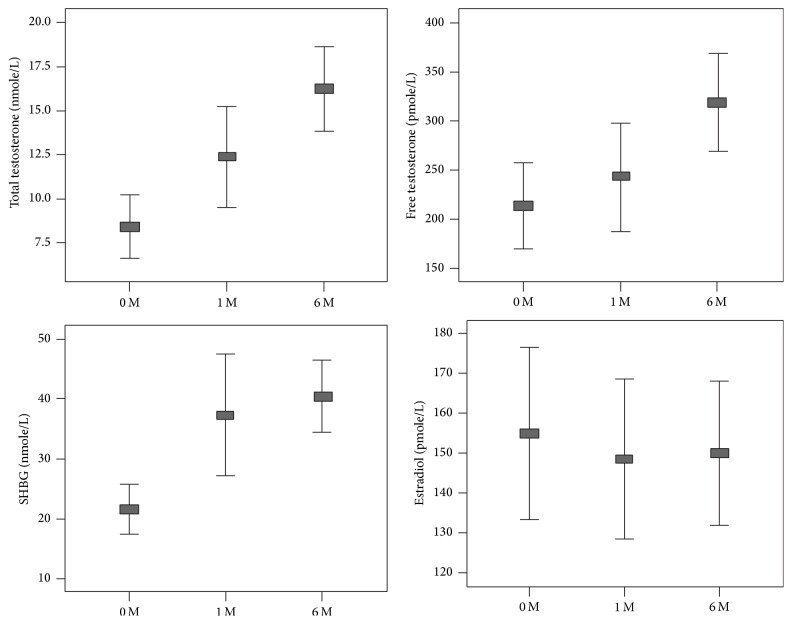
Total testosterone, calculated free testosterone, SHBG, and estradiol at baseline, 1 month and 6 months after bariatric surgery.

Total testosterone increased significantly at 1 month and 6 months after surgery (both *p* ≤ 0.001) (Table 2 and Figure 1). Free testosterone did not change at 1 month after surgery (*p* = 0.352) but increased significantly at 6 months after surgery (*p* ≤ 0.001). SHBG levels increased significantly at 1 month after surgery (*p* ≤ 0.001) but there was no significant change in SHBG from 1 month (39.59 [SD, 21.46]) to 6 months (40.67 [SD, 15.48]) after surgery (*p* = 0.043). There were no changes in estradiol levels either at 1 month or at 6 months after surgery. Leptin levels decreased and adiponectin levels increased significantly after surgery.

The early increase in TT at 1 month after surgery correlated with the increase in SHBG (*r* = 0.472, *p* = 0.042) but not with changes in body weight, BMI, estradiol, adiponectin, or leptin. The correlation between changes in TT and SHBG was also observed at 6 months after surgery (*r* = 0.467, *p* = 0.016) but changes in TT or cFT were not correlated with changes in body weight, BMI, estradiol, adiponectin, or leptin. At 6 months after surgery, there was no significant difference in percentage of weight loss between the patients who normalized TT and cFT and those who did not (25.9% and 22.6% weight loss, resp.).

Hypogonadism score by ADAM questionnaire decreased from 4.6 (SD, 2.5) at baseline to 1.8 (SD, 1.5) at 6 months after surgery (*p* ≤ 0.001).

## 4. Discussion

We found a high prevalence of MOSH (75%) in extremely obese men seeking weight loss surgery. In our study, extremely obese men who underwent bariatric surgery had significant improvements in their testosterone levels and an increase in SHBG levels occurring at 1 month after bariatric surgery. In a recent meta-analysis, weight loss can improve MOSH and weight loss by bariatric surgery is more effective than that by the low-caloric diet in the improvement in testosterone levels [17]. However, most of the previous studies have looked at the changes in sex hormone profiles at 24–52 weeks after bariatric surgery where the weight loss has mitigated.

Remarkably from our study, the increases in TT and SHBG levels occurred early at 1 month after surgery during the caloric restriction phase while most patients can take in less than 1,000 calories per day and the weight loss occurs rapidly. SHBG level is known to be inversely correlated with insulin resistance and is a predictor of the risk of type 2 diabetes [18]. The low SBHG levels seen in obese men at baseline are likely due to the high levels of insulin resistance and the increase in SHBG seen during the first month after bariatric surgery likely reflects the decrease in insulin resistance with rapid weight loss and caloric restriction.

In our study, minimal change in cFT was observed at 1 month but significant increase in cFT levels was evident later at 6 months after surgery. Consistent with previous studies, increase in cFT was seen at more than 6 months after bariatric surgery [12, 19]. Although free testosterone levels did not change during the first month, TT increased along with SHBG suggesting that the testosterone production has already increased. In line with our results, Botella-Carretero et al. [14] have previously shown that increase in testosterone after bariatric surgery was associated with changes in SHBG and insulin resistance calculated by the homeostasis model assessment (HOMA). Unfortunately, we do not have marker of insulin resistance available in our study, but our results could suggest that insulin resistance may play a role in MOSH seen in extremely obese men.

Estradiol levels were measured by immunoassay, rather than a gold standard mass spectrometry, that may be subjected to reduce specificity at low estradiol concentrations in healthy men [20]. Nonetheless, in all of our patients, estradiol levels were high at baseline and remained elevated at 6 months after surgery. We did not find any significant change in estradiol level even though there was a substantial loss in body weight and fat mass. Notably, the average BMI of our patients at 6 months after surgery was 43 kg/m^2^ which is still in the morbidly obese category. Previous studies have shown inconsistent results on the changes in estradiol level after weight loss [17]. Reis et al. [21] reported no significant change in estradiol level whereas Facchiano et al. [22] and Samavat et al. [23] showed reductions in estradiol levels after bariatric surgery. Baseline BMI of patients in those studies were around 43–46 kg/m^2^ which are much lower than baseline BMI in our study (57 kg/m^2^) and this may explain our distinct result. We deem that elevated estradiol level could have an effect but is not the main mechanism in MOSH. Adiponectin increased and leptin decreased after bariatric surgery as expected but we did not find any correlations with the changes in sex hormones profiles.

In our study, only the changes in TT and SHBG were correlated at both 1 month and 6 months after surgery. Although body weight/BMI was known to be inversely correlated with testosterone levels, in our study, the extent of the increase in TT/cFT after surgery did not correlate with the extent of body weight/BMI loss. We believe that MOSH is a complex disorder, driven not only by an increase in body weight but also from insulin resistance and some other mechanisms warranted further study. Together, improvement in TT levels may also facilitate further weight loss in these obese men as previously reported by Saad et al. that treatment with testosterone in hypogonadal men with class III obesity resulted in a significant weight reduction [24].

Hypogonadism score by ADAM questionnaire decreased at 6 months after surgery as testosterone levels have improved. Although ADAM questionnaire is a screening tool to detect androgen deficiency and has some limitations, it is a feasible tool to use in routine clinical practice. A more comprehensive questionnaire for assessing sexual dysfunction and semen analysis may be added to future research in men with MOSH undergoing bariatric surgery.

The strength of our study is that we examined acute changes in sex hormone profiles during the rapid weight loss phase after bariatric surgery and first study to report the prevalence and the reversibility of MOSH in extremely obese Asian men. There are several limitations in our study. First, only extremely obese and young men were included in the study. Secondly, two different surgical procedures were used in the study and we did not have enough power to examine the different in effects of the two procedures. Thirdly, we did not measure gonadotropin levels or examine semen quality. Nonetheless, all patients in our study had improvement in their testosterone levels suggesting the acquired process of their hypogonadism and the reversibility of MOSH with weight loss as documented by other studies. Lastly, we calculated free testosterone using the Vermeulen equation instead of measuring free testosterone by equilibrium dialysis yet the cFT equation was previously reported to have a good correlation with FT levels by direct measurement method [16, 25].

## 5. Conclusions

Hypogonadism is prevalent in extremely obese men and can be reversed with weight loss after bariatric surgery. Increases in TT and SHBG levels occurred early during rapid weight loss at 1 month after bariatric surgery suggesting that insulin resistance plays a major role in MOSH though elevated estradiol level persisted after bariatric surgery. Changes in total testosterone levels tend to be correlated with SHBG levels but not with body weight or adipokines levels.

## Figures and Tables

**Table 1:** Baseline characteristics of participants (*n* = 29).

Age (year)	30.8 ± 8.1

Comorbidities [*n* (%)]	
Hypertension	19 (65.5%)
Diabetes	7 (24.1%)
Dyslipidemia	19 (65.5%)
NAFLD/NASH	20 (69.0%)
Obstructive sleep apnea	29 (100%)

Type of surgery	
RYGB	15 (51.7%)
SG	14 (48.3%)

Body weight (kg)	168.3 ± 35.0

BMI (kg/m^2^)	56.8 ± 11.7

SBP (mmHg)	133 ± 26

DBP (mmHg)	83 ± 12

FPG (mg/dL)	99.0 [89.3–122.8]^*∗*^

HbA1c (%)	6.0 [5.2–6.3]^*∗*^

Total cholesterol (mg/dL)	211 ± 56

HDL (mg/dL)	39 ± 11

Triglyceride (mg/dL)	178 ± 86

LDL (mg/dL)	139 ± 42

Albumin (gm/dL)	3.9 ± 0.1

AST (U/L)	37.1 ± 6.6

ALT (U/L)	55.8 ± 9.5

Data are expressed as means ± SD or number (percentage).

^*∗*^Median [interquartile range].

**Table 2:** Changes in parameters after bariatric surgery and percentage changes from baseline.

	Baseline	Month 1	Month 6
Body weight (kg)	168.3 ± 34.9	146.0 ± 31.7^a^	126.2 ± 25.5^a^
% change	—	−9.4 ± 0.6	−25.1 ± 0.7

BMI (kg/m^2^)	56.9 ± 11.7	50.2 ± 11.0^a^	42.9 ± 9.0^a^
% change	—	−9.9 ± 0.9	−25.7 ± 0.9

Total testosterone (nmol/L)	8.38 ± 4.67	12.60 ± 6.09^a^	15.81 ± 5.95^a^
% change	—	59.8 ± 17.0	126.9 ± 16.6

Calculated free testosterone (pmol/L)	214.0 ± 113.0	245.3 ± 114.7	307.9 ± 121.7^a^
% change	—	19.8 ± 13.2	60.7 ± 10.2

SHBG (nmol/L)	21.59 ± 10.71	39.59 ± 21.46^a^	40.67 ± 15.4^a^
% change	—	98.4 ± 22.0	138.2 ± 23.0

Estradiol (pmol/L)	155.1 ± 56.6	155.2 ± 39.4	149.5 ± 47.1
% change	—	12.0 ± 8.2	2.6 ± 5.7

Adiponectin (*µ*g/mL)	6851.5 ± 4793.5	11942.9 ± 8727.6^a^	12456.2 ± 9160.2^a^
% change	—	60.8 ± 15.1	105.8 ± 26.8

Leptin (ng/mL)	46.21 ± 18.86	32.84 ± 17.72^a^	21.32 ± 11.01^a^
% change	—	−26.9 ± 6.9	−59.6 ± 4.8

Data are expressed as means ± SD.

Compared with baseline values using paired *t*-tests; ^a^ *p* < 0.01.
